# Development of a Search Strategy for an Evidence Based Retrieval Service

**DOI:** 10.1371/journal.pone.0167170

**Published:** 2016-12-09

**Authors:** Gah Juan Ho, Su May Liew, Chirk Jenn Ng, Ranita Hisham Shunmugam, Paul Glasziou

**Affiliations:** 1 Department of Primary Care Medicine, Faculty of Medicine, University of Malaya, Kuala Lumpur, Malaysia; 2 Faculty of Health Sciences and Medicine, Bond University, Queensland, Australia; Youngstown State University, UNITED STATES

## Abstract

**Background:**

Physicians are often encouraged to locate answers for their clinical queries via an evidence-based literature search approach. The methods used are often not clearly specified. Inappropriate search strategies, time constraint and contradictory information complicate evidence retrieval.

**Aims:**

Our study aimed to develop a search strategy to answer clinical queries among physicians in a primary care setting

**Methods:**

Six clinical questions of different medical conditions seen in primary care were formulated. A series of experimental searches to answer each question was conducted on 3 commonly advocated medical databases. We compared search results from a PICO (patients, intervention, comparison, outcome) framework for questions using different combinations of PICO elements. We also compared outcomes from doing searches using text words, Medical Subject Headings (MeSH), or a combination of both. All searches were documented using screenshots and saved search strategies.

**Results:**

Answers to all 6 questions using the PICO framework were found. A higher number of systematic reviews were obtained using a 2 PICO element search compared to a 4 element search. A more optimal choice of search is a combination of both text words and MeSH terms. Despite searching using the Systematic Review filter, many non-systematic reviews or narrative reviews were found in PubMed. There was poor overlap between outcomes of searches using different databases. The duration of search and screening for the 6 questions ranged from 1 to 4 hours.

**Conclusion:**

This strategy has been shown to be feasible and can provide evidence to doctors’ clinical questions. It has the potential to be incorporated into an interventional study to determine the impact of an online evidence retrieval system.

## Introduction

The practice of evidence based medicine (EBM) involves recognizing uncertainties and formulating these as an answerable question, searching for the literature evidence, appraising the evidences, and applying the acquired knowledge to the patient. In daily clinical practice, doctors encounter up to six clinical questions per patient; however, 70% of the questions are left unanswered [1]. Doctors usually seek answers from colleagues since it is easier and faster [2]. They also search for medical information by using their national guidelines which unfortunately are often out of date [3]. These diagnostic or treatment guidelines are often not able to provide the best evidence for patients due to inaccurate and outdated information which may harm patients [3]. Increasing access to clinical evidence improves the adoption of evidence-based practice among primary care physician [4]. This has been shown to have a positive impact on medical decision making which lead to quality patient [5].

Searching medical research databases is often perceived to be as easy as a general internet search [6], and few physicians seek to acquire searching skills themselves [7]. They may not be able to structure their question into an answerable form; answers can be different according to the words that they use to search the database [8]. The lack of quick and easy identification of relevant online literature was also described by Agoritsas and colleagues (2014) [8]. Also, standard search engines (e.g. Google) are not designed to handle the variety of evidence sources that cater for the information needs of physicians. Therefore, those seeking evidence could easily get lost in the sea of information with an average of 75 trials and 11 systematic reviews churned out per day by the industry [9–10]. This problem is further compounded by conflicting information. Conflicting information exist because of the different conclusions arrived from different papers within a similar topic. The possibility of automating several tasks in the evidence-based information retrieval process using informatics has been explored [11] to keep pace with overwhelming amount of research papers.

The support of a clinical evidence search service with the help of librarians has been found to make the task of applying evidence-based practice in clinicians’ daily practice less daunting [4]. Studies on this have looked at participants conducting the search themselves after training from librarians, utilising a librarian assisted services or a combination of both [12–13]. These interventions have demonstrated benefits of their own which include improved searching skills and efficient literature searching [14]. Medical librarians are able to locate satisfactory answers to at least 46% of the questions randomly submitted by primary care physicians [1].

Searching in a combination of databases has been shown to answer a higher proportion of questions [1]. A single database search is known to be inadequate for systematic reviews as the non-inclusion of missed trials would influence the results of the meta-analysis [15–16]. The study also recommended that the Cochrane Library should be searched together with PubMed [15]. Restricting the search to one database would miss many papers and therefore affect the overall results retrieved [17]. A study highlighted the importance of a combination of three databases to achieve a 90% retrieval of relevant literature on the subject areas [18]. Each database has its unique search terms and architecture which makes it challenging to retrieve relevant articles. This makes the process time consuming and a proper search would often require the skills of a medical librarian. It is unlikely that a doctor will be able to have the skill or resources to conduct such a comprehensive search to answer clinical questions for practice.

This paper describes the development and architecture of a search strategy for retrieving literature evidence to answer clinical questions. It is simple, time- and labour-efficient yet comprehensive and uses databases (PubMed, Cochrane Library and TRIP database) that are commonly used by physicians.

## Methods

The search strategy was developed via a series of brainstorming sessions among 4 research team members—2 from the librarian team (RH and AH), the other 2 from the EBM consultant team (SM and CJ) coordinated by the principal investigator (GJ). An initial search plan was conceptualized via expert consensus coupled with the gathering of previous literature.

### Database Selection

The databases chosen were: TRIP database, PubMed and Cochrane as they were recommended by most EBM guides and most highly referred to by physicians [19]. These databases are also available for free (excluding the TRIP database premium version), easily searchable, and self-described as up-to-date. These databases provide centralized evaluation and selection by clinical editors to evaluate the validity of information. Hence, a good mix of comprehensive medical search engine (PubMed), recognised gold standard of evidence-based practice (Cochrane Library) and high-quality filtered pre-appraised source (TRIP database) will be valuable to the development of a search strategy. Full text articles were accessed via digital library of the University of Malaya.

### PICO framework

The PICO framework is known to help searchers achieve relevant results of higher precision [20]. It formats a clinical question in 4 components: population (P), intervention (I), comparison (C) and outcome (O) [21]. It allows better specificity and conceptual clarity to the clinical problem [22]. This is because it dissects the questions into smaller components which are then easier to search [23]. In this study, the use of different combinations of PICO elements in the search strategy were documented.

### Clinical Questions

Six clinical questions were identified by the research team and categorized into the PICO framework as shown in Table 1. These questions were chosen because:

1. They are common acute and chronic illnesses seen by family physicians during their clinical practice.
2. They consist of diagnostic or therapeutic questions which are the most commonly asked question type [24].
3. They represent different patient groups (eg, men, women, infant, senior citizens etc.).

**Table 1:** Six Clinical Questions for Testing Search Strategy.

No.	Question	Types of Question	Population	Intervention	Comparison	Outcome
1	Is ibuprofen more effective than paracetamol in relieving fever in children?	Therapeutic	Child	Ibuprofen	Paracetamol	Reduction of fever
2	Do multiple or single courses of antenatal corticosteroid therapy reduce complications among singleton pregnancies?	Therapeutic	Pre-term labour	Repeated corticosteroid injection	No repeated corticosteroid injection	Premature lungs in new-born
3	Is self-sampling more effective than pap smear in detecting cervical cancer among older women?	Diagnostic	Older women	Self -cervical brush	Pap’s smear	Detection of cervical cancer
4	Is DEXA scan an effective screening tool for detecting osteoporosis in men?	Diagnostic	Men	DEXA scan		Osteoporotic screening
5	What is the most effective antibiotic agent for treating cellulities in patients with diabetes?	Therapeutic	Type 2 Diabetes mellitus and cellulitis	Anti-bacterial agent	Other anti-bacterial agents	Symptom relief, resolution of infection
6	Is acupuncture more effective than physiotherapy in improving mobility among ischaemic stroke patients with right hemiparesis?	Therapeutic	Patients with ischaemic stroke & right hemiparesis	Acupuncture	Physiotherapy	Improvement in mobility

### Search Strategy

The development of our search strategy was intended to cover the first two steps (1. Asking the right questions. 2. Acquiring the evidence) of the 5A’s of EBM [25]. The search strategy was developed by one author (RH) and was continuously reviewed and revised by 2 others (GJ and AH).

The search was conducted under the instruction of a medical librarian (RH) by two research assistants (GJ, AH) who were trained in literature search. To obtain the average time taken to search, each search was repeated three times by different searchers.

Keywords used were dependent on the PICO elements used in different combinations. The search techniques utilised were Boolean operators, truncation, subject headings and filters. Truncation, in health informatics refers to the deliberate shortening of a search term, usually at its root, by the use of wildcard characters to retrieve work variants due to differences in language or tenses [26]. Searches were limited to title and abstract.

When too many (more than 200) articles were retrieved with the strategy used, searches were restricted to randomised controlled trials, systematic reviews & meta-analysis. Repeated, or withdrawn articles (judged on the similarity of title names and content) from each database were not used for analysis. Articles of more than 10 years old were omitted unless there were no systematic reviews found in the recent decade. Answers were considered “satisfactory” if they answered the question using systematic reviews. Articles selected after checking for relevance were combined and summarized.

**Fig 1:**
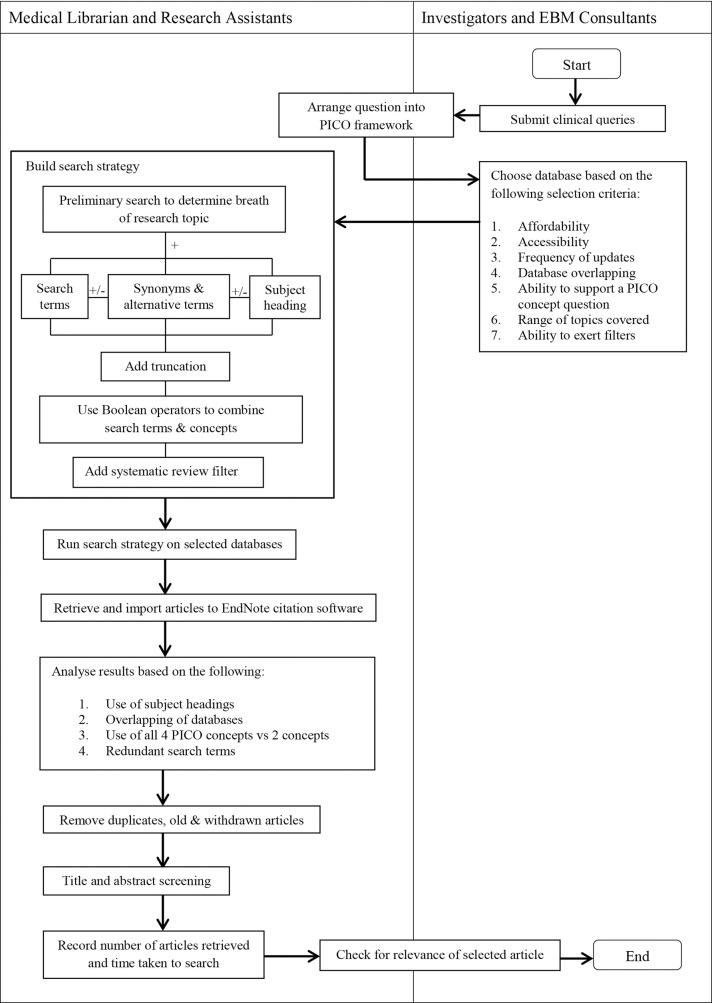
Steps taken to develop the search strategy.

In our strategy, a hierarchical approach was used. Once a systematic review which has the highest level of evidence is obtained, no further search at lower evidence level was attempted. We increased the efficacy of our search strategy by reducing common errors [27] such as spelling errors or using wrong line numbers. Some search methods recommended for conducting a systematic review were followed but modified to achieve a pragmatic concise search. We were not attempting to replicate a full systematic review. This involved finding a balance between the broadness, relevance and the amount of free-text terms used. The process flow (Fig 1) of the steps taken to develop the search is described.

### Selection of articles

Title selection was conducted by 2 reviewers of the team and any disagreement was resolved by a consensus-based discussion or by the decision of a third independent reviewer. Reasons of article not being selected were recorded in EndNote. A screenshot of each strategy were taken for documentation.

### Outcomes

The primary outcome was the answers to questions retrieved from each of the databases. Secondary outcomes include consistency of the answers, time taken to search and overall quality of retrieval performance. Issues that arose from the search process were identified.

## Results

### Time taken to answer questions

It took an average of 147 minutes with the fastest time being 135 minutes from structuring a question to answering a question. The actual mean time spent on search was 48.6 minutes (Step 1 to 5). The rest of the time was spent on screening and checking for relevance and quality of the articles. The amount of time taken for this depended on the number of articles that were retrieved by the search strategy; and needed to be checked and screened for relevance. Answering all six questions took 14 hours as shown in Table 2.

**Table 2:** Time taken for the search strategy to be executed in minutes.

	Time taken (min.)	Question 1			Question 2			Question 3			Question 4			Question 5			Question 6			Mean time (min.)
		Cochrane	PubMed	TRIP	Cochrane	PubMed	TRIP	Cochrane	PubMed	TRIP	Cochrane	PubMed	TRIP	Cochrane	PubMed	TRIP	Cochrane	PubMed	TRIP	
1	Formulation of Answerable Question in PICO	5			6			4			5			6			4			5
2	Characterization of Question	3			2			4			2			4			4			3.2
3	Formulation of Search Terms	11			15			16			9			15			8			12.3
4	Preliminary Search	15			17			21			15			28			15			18.5
5	Application of Search Strategy to Retrieve Articles	10	14	9	9	12	7	7	12	5	8	10	10	9	12	5	8	17	9	9.6
6	Title and Abstract Screening	14			15			13			11			12			11			12.7
7	Checking for Article Relevance^a^	42 (7)			50 (7)			40 (5)			46 (7)			36 (4)			70 (12)			47.3(7)
8	Summarization of Evidence	12			18			15			27			13			32			19.5
	Total	135			151			137			143			140			178			147.3

^a^ Number of articles appraised after removing duplications amongst databases are indicated in brackets

### Database used

Using our search strategy, PubMed retrieved the highest number of articles as seen in Table 2, which explains the longer time taken to screen through the articles from PubMed followed by Cochrane Library and TRIP database respectively. PubMed retrieved many reviews but these were mostly found to be of narrative or descriptive nature and not systematic reviews despite using the Systematic Review filter.

Compared to PubMed and TRIP database, the Cochrane Library is less effective in retrieving answers to diagnostic clinical questions (Question 3 and 4). This can be seen in Question 4 where Cochrane retrieved a total of 146 papers but only three were relevant studies after title selection. Many papers were retrieved when searching for a diagnostic paper in Cochrane Library but only a small percentage was relevant.

### Findings from search strategy used

#### PICO framework

The effectiveness and efficiency of the search execution was constantly improved after each search. During article screening, many unrelated papers were found during our search which requires a significant amount of time to sift through this information. This is because using two PICO elements increased the search sensitivity but reduced its specificity. However, using all four PICO elements in the first three questions retrieved too few results as the searches were too narrow. The search was switched to using a maximum of three elements with ‘Population’ and ‘Intervention’ being the two most important elements used. An example of the search strategy executed on Question 1 is shown in Table 3. It describes the three strategies attempted to differentiate the results obtained when using free text words, index terms and a combination of both.

**Table 3:** The search strategy executed in Question 1.

Database used	Cochrane Library^1^			PubMed^1^			TRIP Database	
Strategies used	Indexed search terms	Free text words	Combination of free text words and indexed terms	Indexed search terms	Free text words	Combination of free text words and indexed terms	Free text words^2^	Free text words
Participant/ Patient^3^	• MeSH descriptor: [Child] explode all trees, • MeSH descriptor: [Infant] explode all tree	• Child, • Children • Infant • infants	• MeSH descriptor:[Child] explode all trees • MeSH descriptor: [Infant] explode all tree • Child • Children • Infant • infants	• “Children” • [MeSH}	• child, • children, • infant*	• “Children” [MeSH] • Child • Children • infant*	• Child* • infant*	• children • infant
Intervention^4^	• MeSH descriptor: [Acetaminophen] explode all trees	• Acetaminophen • Panadol • Tylenol • paracetamol	• MeSH descriptor: [Acetaminophen] explode all trees • Acetaminophen • Panadol • Tylenol • paracetamol	• "Acetaminophen [MeSH]	• Acetaminophen • Panadol • Tylenol • paracetamol	• "Acetaminophen[MeSH] • Acetaminophen • Panadol • Tylenol • paracetamol	• paracetamol • panadol, • acetaminophen	• paracetamol • acetaminophen
Comparison^5^	• MeSH descriptor:[Ibuprofen] explode all trees	• Ibuprofen • Advil • Motrin • brufen	• MeSH descriptor: [Ibuprofen] explode all tree • Ibuprofen • Advil • Motrin • brufen	• “Ibuprofen” [MeSH]	• ibuprofen • advil • motrin • brufen	• “Ibuprofen” [MeSH] • Ibuprofen • Advil • Motrin • brufen	• ibuprofen • advil • motrin • brufen	• ibuprofen
Number of Systematic Reviews Retrieved	1	93	5	23	51	56	5	5
Articles chosen after Title Screening	1	11	2	10	12	17	4	4
Articles chosen after Abstract Screening	1	5	1	7	7	3	3	3

^1^ Cochrane and PubMed both shared index terms from the same source, which is also known as Medical Subject Heading (MeSH).

^2^ Search terms on TRIP database was not indexed due to the lack of indexing system in the database when the search was conducted. The strategy was carried out to explore the amount of answers retrieved with and without the use of alternative terms as well as truncation. The amount of answers retrieved is the same.

^3,4,5^All search terms in one element are linked with Boolean operator “OR”, after which search terms between different elements are linked by Boolean operator “AND”

### Issues Identified during Development of Search Strategy

#### Search term redundancy

When a low number of systematic reviews were found in that database, the strategy was modified accordingly to increase search yield. Determining the relevance of the search term can be subjective as retrieving more papers using more search terms does not add value to the end result. For example when answering Question 5 in TRIP Database, using the search term ‘osteoporosis’ was redundant since DEXA is mostly use for osteoporosis screening. Without using the term ‘osteoporosis’, seven more systematic reviews were retrieved, but these papers were deemed irrelevant after title screening. This means that ‘DEXA’ is a more specific word than “osteoporosis”. Using only the most important search terms improves specificity to the answers retrieved and saves time too.

#### Using both subject headings and free-text terms

**Fig 2:**
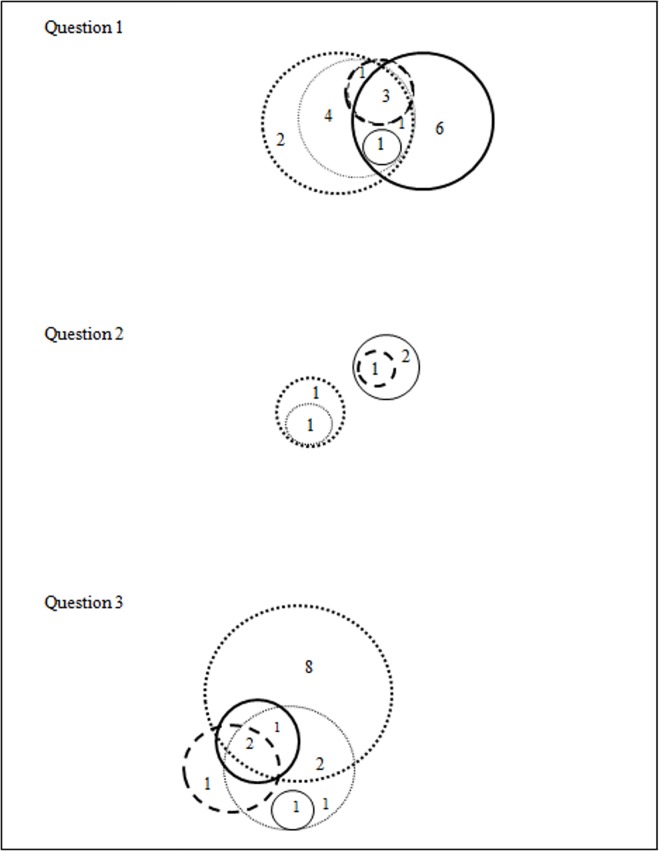
Intersection of selected databases and the use of subject headings. Cochrane Library; MeSH terms only (designated with thin straight line), Cochrane Library; free text words only (designated with thick straight line), PubMed; MeSH terms (designated with thin dotted line), PubMed; free text words only (designated with thick dotted line), TRIP Database; free text words only (designated with thin dashed line), TRIP Database; free text words with alternative terms (designated with thick dashed line).

PubMed and Cochrane have their own indexing language which is MeSH. This allowed standardardisation of key terms. Although the TRIP Database has a synonym function, it was found to be less adaptable to our search strategy because the database does not have its own controlled vocabulary. When search terms (Fig 2) were entered together with synonyms or alternative terms, more relevant results were obtained.

During the search process, we managed to identify some articles that are only captured when using a combination of text words and index terms. Interestingly, two completely different sets of results emerged when using free text words or index terms separately. In 2 questions, up to 3 papers were not captured when search was carried out using just free text words alone. A combination of both free text words and index terms managed to retrieve extra papers that would not have been found if searched separately.

#### Alternative Terms

No results were retrieved when using just index terms of ‘diabetes’ and ‘cellulitis’ in Question 5. However, some results were retrieved when using the word ‘bacterial skin infection’ together with ‘diabetes’. In Question 4, when searching in TRIP Database using only ‘osteoporosis men’ and ‘DEXA scan’ without the alternative term ‘absorptiometry’, no results were obtained. This makes ‘absorptiometry’ an important alternative term of ‘DEXA scan’ to retrieve relevant articles. This shows the importance of including related terms or synonyms of the search term when conducting an effective search.

#### Evidence found

We found systematic review answers to all 6 questions. There were however similar systematic reviews that arrived at different conclusions. For example, in Question 2, there were 2 papers that are a year apart which presented different conclusions.

#### Overlap of databases

There was database overlap (Fig 2) although the patterns of intersection were unclear for the questions tested.

#### Documentation of process

PubMed and Cochrane Library allowed complete export of citation information to the EndNote citation software. TRIP Database does not provide the full reference details of the study fully when imported to EndNote. Abstract are not accessible when reading TRIP Database. Certain information of the study such as authors has to be entered manually. This makes documentation a laborious process.

## Discussion

One of the major findings in our study is that a higher number of systematic reviews were obtained using a 2 PICO element search compared to a 4 element search. Answers to all 6 questions using the PICO framework were found. We also found that using a combination of both text words and MeSH terms is a more optimal choice of search. Despite searching using the Systematic Review filter, many non-systematic reviews or narrative reviews were found in PubMed. The duration of search and screening using this strategy ranged from 1 to 3 hours There was poor overlap between outcomes of searches using different databases. Due to the lack of overlap between databases, the difference in coverage and timeliness of content updating, using all 3 databases would be recommended to avoid missing articles. We suggest a process of de-duplication after exporting citations into the EndNote software. De-duplication refers to the removal of duplicated articles. This can be achieved by stacking identical articles according to the title name to EndNote, removing the similar papers and kept only one of each.

This study has shown that this strategy is feasible for use. The mean time taken by the search itself for one question was 48.6 minutes; this excludes the steps on checking for relevance and summarization of evidence. It is possible to eliminate these 2 time-consuming steps in a search service as physicians who submit a clinical query might prefer to check and summarize the papers themselves. It is likely that they would be better able to determine the relevance and applicability of the selected articles to their patients or clinical practice. Searches were carried out by human subjects using computers, which might cause latency in response time compared to automated computer searches. Structuring a clinical question correctly into a PICO format help saves time. It dissects the question into components and restructures it into a clearer and more specific question to yield faster results [23]. Time delay usually occurs when seeking clarification to refine the question. These are expected if physicians were to submit queries to us. We therefore recommend teaching physicians how to ask a clinical question in a structured way to provide a faster search [28]. The use of advanced search techniques and filters may potentially reduce the time taken to search. However, these features are subject to the performance of the database used [29].

One way of improving PICO search yield is to include indexing terms and synonyms related to the PICOs. The precision of the search can be increased by adding more related terms. Another challenge to retrieve evidence was to identify relevant search terms and their synonyms. There was a lot of trial and error involved in the process. This is done by adding and removing search terms or their synonyms to determine relevance based on the amount and quality of the results retrieved. This process is repeated for the included PICO elements. Contrary to popular studies in which formulating a question using four aspects of the PICO were encouraged, a study [19] has shown that clinical questions which can answer all four PICO elements are rare and the population (P) and intervention (I) are the most frequent elements which needed to be addressed.

Although the use of free text terms and subject headings have not been properly surveyed, our study confirms that new and updated articles are best located using a combination of index term and free text words to enhance search performance as many new articles have yet to be indexed. The indexing pattern in TRIP database cannot be assessed as TRIP still does not have its own controlled vocabulary [30]. Further assessments are still needed to evaluate the database properly for future inclusion of its own indexing system.

As every database is structured in its own unique architecture, it is essential to tailor the search strategy to the individual database [26]. The structure of the search strategy however remains largely the same despite adapting the strategy into the different databases. Cochrane reviews are included in PubMed, but the results retrieved were different. This further shows the importance of including both databases as well as TRIP Database to complement each other for a comprehensive search. The differences in the nature of coverage from each database necessitate using all three databases to achieve a more comprehensive search result.

A comprehensive search to a clinical question may result in increased uncertainty when evidence obtained is conflicting. As most physicians only look at the abstract, discussion and conclusions, the content written on these systematic reviews can be overlooked and might influence physicians’ clinical decision making. This reaffirms the importance of appraising skills among physicians. One way to address this may be to include an appraisal of the evidence into the service. However, this would render the service less feasible as it requires additional resources such as time, labour and skills.

### Limitation and Recommendation of Study

The main limitation of the study is the limited number of clinical questions used for strategy development. More questions covering other medical conditions and treatment would have likely have revealed greater issues. Although these questions were derived from clinical cases seen by primary care physicians, they are not fully representative of actual patients as they address a single clinical concern and this may not give a complete or true picture of the complexity of primary care patients.

In our study we did not resort to exhaustive searching, citation tracking or hand searching which would have allowed us to retrieve an even higher number of articles because this will compromise the time efficiency of our search. Such an approach would have been unfeasible. There is also the concept of bibliographic futility, where searching should be discontinued when available data showed that further search will not affect the overall result of retrieval [31]. However, this was not explored in this study.

Although the study was aimed at primary care physicians, the search strategy may also be helpful in answering the clinical questions of other healthcare practitioners. Further research is recommended to determine the usability of the search strategy in different healthcare practitioners.

## Conclusion

Based on the strategy developed, we found that searching for systematic reviews in PubMed, Cochrane Library and TRIP Database using a combination of free text words and index terms with the two most important PICO elements provided higher quality performance of evidence retrieval. Our strategy is useful in searching for articles in which the topics have been rigorously researched on. We plan to pilot test this strategy by studying the usability and feasibility of an evidence retrieval service for primary care physicians.

## Supporting Information

S1 Table Search strategy for Question 1 using all PICO elements without subject headings. (DOCX)

S2 Table Search strategy for Question 1 using 3 PICO elements with subject headings. (DOCX)

S3 Table Search strategy for Question 2 using 3 PICO elements without subject headings. (DOCX)

S4 Table Search strategy for Question 2 using 2 PICO elements with subject headings. (DOCX)

S5 Table Search strategy for Question 3 using all PICO elements without subject headings. (DOCX)

S6 Table Search strategy for Question 3 using 2 PICO elements with subject headings. (DOCX)

S7 Table Search strategy for Question 4 using 2 PICO elements with subject headings. (DOCX)

S8 Table Search strategy for Question 5 using 2 PICO elements with subject headings. (DOCX)

S9 Table Search strategy for Question 6 using 2 PICO elements with subject headings. (DOCX)
