# Supplementary material for: Development of a Search Strategy for an Evidence Based Retrieval Service
Source: PLoS One. 2016 Dec 9;11(12):e0167170. doi: 10.1371/journal.pone.0167170 (PMC5147858; doi:10.1371/journal.pone.0167170)
Supplement: S1 Table — (DOCX) [file pone.0167170.s001.docx]

|  | **Cochrane Library** | **PubMed – Clin. Queries** | **TRIP** |
| --- | --- | --- | --- |
| P | child***,** infant*, pediatrics**,** paediatrics | | |
| I | ibuprofen, advil, motrin, brufen | | |
| C | paracetamol, acetaminophen, panadol | | |
| O | fever, febrile, “high temperature” | | |
| Number of SR Retrieved | 5 | 25 | 3 |
| Articles chosen based on title | 1 | 16 | 3 |
| Articles chosen based on abstract | 1 | 3 | 1 |

**Supporting Information 1**

SI Table. **Search strategy for Question 1 using all PICO elements without subject headings**
