# Supplementary material for: Development of a Search Strategy for an Evidence Based Retrieval Service
Source: PLoS One. 2016 Dec 9;11(12):e0167170. doi: 10.1371/journal.pone.0167170 (PMC5147858; doi:10.1371/journal.pone.0167170)
Supplement: S2 Table — (DOCX) [file pone.0167170.s002.docx]

**Supporting Information 2**

S2 Table. **Search strategy for Question 1 using 3 PICO elements with subject headings**

|  | **Cochrane Library** | | **PubMed – SR Filter** | | **TRIP** | |
| --- | --- | --- | --- | --- | --- | --- |
| P | MeSH descriptor: [Child] explode all trees, MeSH descriptor: [Infant] explode all tree | child or children, infant or infants | “Children”  [MeSH] | child, children, infant* | Child*, infant* | children, infant |
| I | MeSH descriptor: [Acetaminophen] explode all trees | acetaminophen, panadol, tylenol, paracetamol | "Acetaminophen"  [MeSH] | acetaminophen, panadol, tylenol, paracetamol | paracetamol, panadol, acetaminophen | paracetamol, acetaminophen |
| C | MeSH descriptor: [Ibuprofen] explode all trees | ibuprofen, advil, motrin, brufen | “Ibuprofen”  [MeSH] | ibuprofen, advil, motrin, brufen | ibuprofen, advil, motrin, brufen | ibuprofen |
| Number of SR Retrieved | 1 | 93 | 23 | 51 | 5 | 5 |
| Articles chosen based on title | 1 | 11 | 10 | 12 | 4 | 4 |
| Articles chosen based on abstract | 1 | 5 | 7 | 7 | 3 | 3 |
