# Supplementary material for: Development of a Search Strategy for an Evidence Based Retrieval Service
Source: PLoS One. 2016 Dec 9;11(12):e0167170. doi: 10.1371/journal.pone.0167170 (PMC5147858; doi:10.1371/journal.pone.0167170)
Supplement: S3 Table — (DOCX) [file pone.0167170.s003.docx]

**Supporting Information 3**

S3 Table. **Search strategy for Question 2 using 3 PICO elements without subject headings**

|  | **Cochrane Library** | **PubMed – Clin. Queries** | **TRIP** |
| --- | --- | --- | --- |
| P | pregnancy (pre-term labour), obstetric labor, premature premature labor, premature obstetric labor, obstetric labor complications, preterm labor | | |
| I | dexamethasone, dexamethasone injection, dexamethasone isonicotinate, decadron | | |
| O | premature lungs, premature birth, pregnancy complications, obstetric labor complications | | |
| Number of SR Retrieved | 18 | 9 | 0 |
| Articles chosen based on title | 4 | 6 | 0 |
| Articles chosen based on abstract | 3 | 4 | 0 |
