# Supplementary material for: Development of a Search Strategy for an Evidence Based Retrieval Service
Source: PLoS One. 2016 Dec 9;11(12):e0167170. doi: 10.1371/journal.pone.0167170 (PMC5147858; doi:10.1371/journal.pone.0167170)
Supplement: S4 Table — (DOCX) [file pone.0167170.s004.docx]

**Supporting Information 4**

S4 Table. **Search strategy for Question 2 using 2 PICO elements with subject headings**

|  | **Cochrane Library** | | **PubMed – SR Filter** | | **TRIP** | |
| --- | --- | --- | --- | --- | --- | --- |
| P | MeSH descriptor: [Obstetric Labor, Premature] explode all tree | premature obstetric labo*r | “Obstetric Labor, Premature” [MeSH] | premature obstetric labo* | pregnancy (pre-term labour), obstetric labor, premature, premature labor , premature obstetric labor, obstetric labor complications, preterm labor | premature obstetric labo* |
| I | MeSH descriptor: [Dexamethasone] explode all trees | dexamethasone, corticosteroid*, glucocorticoid* | “Dexamethasone” [MeSH],“Glucocorticoids” [MeSH] | dexamethasone, corticosteroid*, glucocorticoid* | dexamethasone, corticosteroid*, glucocorticoid* | dexamethasone, corticosteroid*, glucocorticoid* |
| Number of SR Retrieved | 1 | 74 | 18 | 35 | 0 | 20 |
| Articles chosen based on title | 1 | 4 | 5 | 11 | - | 5 |
| Articles chosen based on abstract | 0 | 3 | 4 | 8 | - | 1 |
