# Supplementary material for: Development of a Search Strategy for an Evidence Based Retrieval Service
Source: PLoS One. 2016 Dec 9;11(12):e0167170. doi: 10.1371/journal.pone.0167170 (PMC5147858; doi:10.1371/journal.pone.0167170)
Supplement: S5 Table — (DOCX) [file pone.0167170.s005.docx]

**Supporting Information 5**

S5 Table. **Search strategy for Question 3 using all PICO elements without subject headings**

|  | **Cochrane Library** | **PubMed – Clin. Queries** | **TRIP** |
| --- | --- | --- | --- |
| P | older women, aged, frail elderly, frail older adults | | |
| I | self-cervical brush, self-sampling, cervix brush, brush-based self-sampling | | |
| C | pap smear, papanicolaou test, papanicolaou smear | | |
| O | cervical cancer, uterine cervical neoplasms, female urogenital diseases, cervical cancer, cervix cancer, cervix neoplasms, uterine cervical cancer | | |
| Number of SR Retrieved | 4 | 0 | 1 |
| Articles chosen based on title | 1 | 3 | 0 |
| Articles chosen based on abstract | 0 | 0 | 0 |
