# Supplementary material for: Development of a Search Strategy for an Evidence Based Retrieval Service
Source: PLoS One. 2016 Dec 9;11(12):e0167170. doi: 10.1371/journal.pone.0167170 (PMC5147858; doi:10.1371/journal.pone.0167170)
Supplement: S6 Table — (DOCX) [file pone.0167170.s006.docx]

**Supporting Information 6**

S6 Table. **Search strategy for Question 3 using 2 PICO elements with subject headings**

|  | **Cochrane Library** | | | **PubMed – SR Filter** | | | **TRIP** | |
| --- | --- | --- | --- | --- | --- | --- | --- | --- |
| I | MeSH descriptor: [Papanicolaou Test] explode all trees | pap* smear*, pap* test* | | “Papanicolaou Test” [MeSH] | papanicolaou test*, papanicolaou smear*  pap test*, pap smear*, pap* test*, pap* smear* | | pap smear, papanicolaou test, papanicolaou smear | pap smear |
| C | HPV self-sampling, self-cervical brush, cervix brush, brush-based self-sampling or brush sampling | | | HPV self-sampling, HPV self sampling, HPV self-collection, HPV self collection,  self-cervical brush*, cervix brush*, brush-based self-sampling*, brush sampling* | | | HPV self-sampling brush, self-cervical sampling | HPV self-sampling brush |
| Number of SR Retrieved | 0 | | 104 | 1 | | 3 | 6 | 3 |
| Articles chosen based on title | - | | 3 | 1 | | 2 | 4 | 1 |
| Articles chosen based on abstract | - | | 2 | 1 | | 2 | 3 | 1 |
