# Supplementary material for: Development of a Search Strategy for an Evidence Based Retrieval Service
Source: PLoS One. 2016 Dec 9;11(12):e0167170. doi: 10.1371/journal.pone.0167170 (PMC5147858; doi:10.1371/journal.pone.0167170)
Supplement: S7 Table — (DOCX) [file pone.0167170.s007.docx]

**Supporting Information 7**

S7 Table. **Search strategy for Question 4 using 2 PICO elements with subject headings**

|  | **Cochrane Library** | | **PubMed**  **SR Filter** | | **TRIP** | | |
| --- | --- | --- | --- | --- | --- | --- | --- |
| P | men OR male | | men OR male | | osteoporosis men | | men |
| I | MeSH descriptor: [Absorptiometry, Photon] explode all trees | DEXA scan, DXA scan, Bone Density Scan, dual-energy x-ray absorptiometry | scans, dexa [MeSH terms] | DEXA scan | dexa scan OR absorptiometry | dexa scan | dexa scan OR absorptiometry |
| Number of SR Retrieved | 5 | 143 | 88 | 92 | 3 | 0 | 8 |
| Articles chosen based on title | 1 | 3 | 13 | 13 | 2 | 0 | 2 |
| Articles chosen based on abstract | 0 | 0 | 5 | 5 | 1 | 0 | 1 |
