# Supplementary material for: Development of a Search Strategy for an Evidence Based Retrieval Service
Source: PLoS One. 2016 Dec 9;11(12):e0167170. doi: 10.1371/journal.pone.0167170 (PMC5147858; doi:10.1371/journal.pone.0167170)
Supplement: S8 Table — (DOCX) [file pone.0167170.s008.docx]

**Supporting Information 8**

S8 Table. **Search strategy for Question 5 using 2 PICO elements with subject headings**

|  | **Cochrane Library** | | **PubMed - SR Filter** | | | **TRIP** | |
| --- | --- | --- | --- | --- | --- | --- | --- |
| P | MeSH descriptor: [Diabetes Mellitus, Type 2] explode all trees, MeSH descriptor: [Cellulitis] explode all trees | (diabetes mellitus type 2 OR non-insulin dependent diabetes) AND (cellulitis OR bacterial skin infection) | diabetes mellitus [MeSH terms] AND celluliti [MeSH terms] | (diabetes mellitus OR non-insulin dependent diabetes) AND (bacterial skin infection OR cellulitis) | diabetes skin infection | diabetes mellitus AND cellulitis | (diabetes mellitus type 2 OR non-insulin dependent diabetes) AND (cellulitis OR bacterial skin infection) |
| I | MeSH descriptor: [Anti-Bacterial Agents] explode all trees | antibiotics OR anti-bacterial agent | antibiotics [MeSH terms] | antibiotics OR anti-bacterial agent | antibiotics | antibiotics | antibiotics OR anti-microbial agent |
| Number of SR Retrieved | 0 | 80 | 0 | 0 | 16 | 13 | 49 |
| Articles chosen based on title | 0 | 6 | 0 | 0 | 6 | 2 | 3 |
| Articles chosen based on abstract | 0 | 2 | 0 | 0 | 2 | 0 | 0 |
