# Supplementary material for: Development of a Search Strategy for an Evidence Based Retrieval Service
Source: PLoS One. 2016 Dec 9;11(12):e0167170. doi: 10.1371/journal.pone.0167170 (PMC5147858; doi:10.1371/journal.pone.0167170)
Supplement: S9 Table — (DOCX) [file pone.0167170.s009.docx]

**Supporting Information 9**

S9 Table. **Search strategy for Question 6 using 2 PICO elements with subject headings**

|  | **Cochrane Library** | | **PubMed - SR Filter** | | | | **TRIP** | | |
| --- | --- | --- | --- | --- | --- | --- | --- | --- | --- |
| P | MeSH descriptor: [Stroke] explode all trees | stroke  OR brain attack OR brain infarction OR cerebrovascular injury OR cerebrovascular insult | stroke [MeSH terms] | stroke [MeSH] | stroke | stroke OR brain attack OR brain infarction OR cerebrovascular  injury OR  cerebrovascular  insult | stroke AND hemiparesis | stroke | stroke OR  brain attack OR  brain infarction OR cerebrovascular injury OR cerebrovascular  insult |
| I | MeSH descriptor: [Acupuncture] explode all trees | acupuncture | acupuncture [MeSH terms] | acupuncture [MeSH] | acupuncture | | acupuncture | | |
| Number of SR Retrieved | 2 | 3 | 30 | 3 | 56 | 58 | 0 | 22 | 22 |
| Articles chosen based on title | 2 | 0 | 11 | 3 | 17 | 17 | 0 | 11 | 11 |
| Articles chosen based on abstract | 1 | 0 | 3 | 2 | 3 | 3 | 0 | 10 | 10 |
